# Selection for Resilience in Livestock Production Systems

**DOI:** 10.3390/ijms252313109

**Published:** 2024-12-06

**Authors:** Houda Laghouaouta, Lorenzo J. Fraile, Ramona N. Pena

**Affiliations:** Agrotecnio-CERCA Center, Department of Animal Science, University of Lleida, 25198 Lleida, Catalonia, Spain; houda.laghouaouta@udl.cat (H.L.); lorenzo.fraile@udl.cat (L.J.F.)

**Keywords:** genetic background, quantification, general resilience, disease resistance, selective breeding

## Abstract

Selective breeding for improved animal resilience is becoming critical to increase the sustainability of production systems. Despite the existence of a genetic component for resilience, breeding for improved resilience has been limited by the absence of a consensus on its definition and quantifying method. In this work, we provide a review of (i) the definition of resilience and related concepts such as robustness, resistance, and tolerance; (ii) possible quantifying methods for resilience; (iii) its genetic background; and (iv) insights about its improvement through selective breeding. We suggest that a resilient animal may be defined as an individual that is able to cope with a perturbation(s) and rapidly bounce back to normal functioning if altered. Furthermore, since challenging conditions lead to trade-offs and, consequently, deviations between basic physiological functions, we suggest using these deviations as indicators for resilience. These resilience indicators may also be used as proxies to study the genetic determinism and background of resilience in livestock species. Finally, we discuss possible strategies to improve resilience and review the implementation of associated genetic markers for resilience indicators in selection schemes.

## 1. Introduction

Currently, animal breeding must cope with the challenge of providing enough food for the continuously increasing population worldwide. There is no doubt that selective breeding, along with proper herd management, suitable preventive medicine programs, feed quality, and precision livestock farming technologies, has successfully increased the productivity of farming in pigs [1], chicken [2,3], and dairy cattle [4]. However, selective breeding for enhanced production has resulted in fitness constraints and adversely affected reproductive performance and susceptibility under challenging conditions [5,6,7]. For instance, selective breeding for enhanced productivity led to a higher susceptibility to mastitis [8] and lower reproductive performance [9] in dairy cattle and a higher susceptibility to challenges in pigs [10] and poultry [6,11]. These drawbacks occurred because the focus of breeding programs was initially limited to production traits, and reproduction and health were considered, at the time, secondary traits which were not included in selection schemes [12]. Furthermore, the genetic antagonism between production and health status [10] was not taken into consideration. Moreover, animals are typically selected in high-health-status environments with strict biosecurity measures such as nuclei and genetic multipliers [13]. In contrast, at the bottom of the selection pyramid, these animals are reared in commercial farms undergoing a wide range of internal and external challenges. Hence, it is necessary to select animals not only for increased productivity but also for their ability to withstand challenges and express their maximum genetic potential [12,13,14]; such animals are termed “**resilient**” (see Box 1). Selection for such productive and resilient animals would reduce economic losses and increase the profitability and sustainability of production systems [15]. Notably, implementing animal resilience in breeding programs is needed since the current objective involves not only meeting consumers’ demand in terms of products but also addressing arising concerns about animal welfare and well-being while implementing one-health strategies in production management that must include a reduction in antimicrobial consumption. The study of resilience and its possible implementation in breeding programs have been limited due to the complexity of this trait. There is no consensus on the definition of resilience or how to measure it. The objective of this work is to provide a review of previous research and current literature on (i) the definition of resilience; (ii) possible quantifying methods; (iii) genetic backgrounds and determinism; and (iv) insights about potential improvements through selective breeding.

Box 1 **Definition of animal resilience and some related concepts**

**Resilient animal:** an individual that can cope with a perturbation(s); even if its life functions are slightly altered, it can rapidly bounce back to normal functioning.

**Robust animal:** a resilient individual that is able to express its potential production in a wide range of environments.

**Resistant animal:** an individual that can limit and control a pathogen load upon infection.

**Tolerant animal:** an individual whose life functions are minimally affected by a pathogen load.

## 2. Defining Animal Resilience

The concept of resilience has been used in different fields, from psychology [16] to ecology [17] and livestock [18]. Within the latter field, there are multiple definitions for resilience in the scientific literature. Therefore, the aim of this Section is to provide an overview of how resilience and other related concepts, such as robustness, resistance, and tolerance, have been defined and to offer a potential consensus.

Defining **resilience** is an essential first step for its quantification and potential improvement through genetic selection. Most of the given definitions for resilience include one of the following two key elements (Box 1): (i) an animal’s response during a challenge and/or (ii) an animal’s ability to recover after a challenge [18,19,20,21,22].

**Figure 1:**
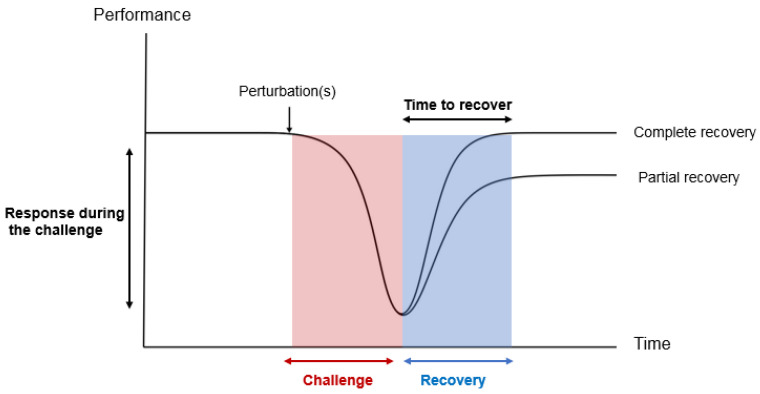
Illustration of resilience responses after a perturbation.

In other words, a resilient animal can be defined as an individual that is able to cope with perturbation(s); although its life functions might be altered, it can rapidly bounce back to normal functioning (Figure 1).

Resilience is, therefore, always linked to a challenge, which may be a pathogen, a social effect, transport, or any possible perturbation that may occur during the productive life of the animal [20]. In some species, these challenges can be cyclical, such as the metabolic stress associated with the start of lactation or seasonal heat stress [23]. These challenges allow us to distinguish between two concepts: disease resilience and general resilience [20]. **Disease resilience** refers to an animal’s response against a specific pathogen. In contrast, **general resilience** refers to an animal’s response to all perturbations that may occur during a period. Notably, general resilience covers multiple facets of animal resilience, including disease resilience. Indeed, it is not always easy to distinguish disease resilience from general resilience since animals may be initially challenged by a single pathogen, but exposed animals may be more susceptible to other perturbations involving other microorganisms or not. For instance, stress increases animals’ susceptibility to other perturbations [24]. Stress hormones, such as glucocorticoids, may affect the immune response and consequently increase susceptibility to perturbations [25]. Accordingly, even mild challenges such as attenuated vaccine administration can initiate the immune response and lead to shifts in energy allocation [26]. Moreover, it is important to point out that we can also distinguish individual resilience (either specific disease resilience or general resilience) from herd resilience, which captures how a disease can be spread in an animal population depending on the animals present in that herd [27].

Another concept related to resilience is **robustness** (Box 1). Similarly to resilience, robustness is not consistently defined, and both concepts, resilience and robustness, are often interchangeably used. Nevertheless, two main differences may be highlighted between them: the type of environmental constraint and the duration of a disturbance. According to some authors, resilience is linked to the ability of an animal to cope with challenges in a given environment, whereas robustness relates to the ability of an animal to cope with challenges in a wide range of environments [5,20,28]. Moreover, resilience usually refers to short-term challenges (such as a sudden change in diet or an infectious outbreak), whereas robustness is related to cyclical and persistent challenges (such as those imposed by seasonal heat stress or by persistent cyclical infections in farms) [20,29]. Another possible difference between resilience and robustness pertains to the performance and productivity of an animal. In contrast to resilience, the definitions of robustness often include terms related to productivity such as “production potential” [20], “high production potential” [5], and “perform well” [14]. Moreover, resilience was included in the definition of robustness given by Knap [5], which suggests that a robust animal is a resilient individual that is able to maintain high productivity in a number of environments, while a resilient animal is not necessarily robust. However, both concepts are often interchangeably used in the literature, especially robustness and an animal’s general resilience, given the large overlap between these two concepts.

**Figure 2:**
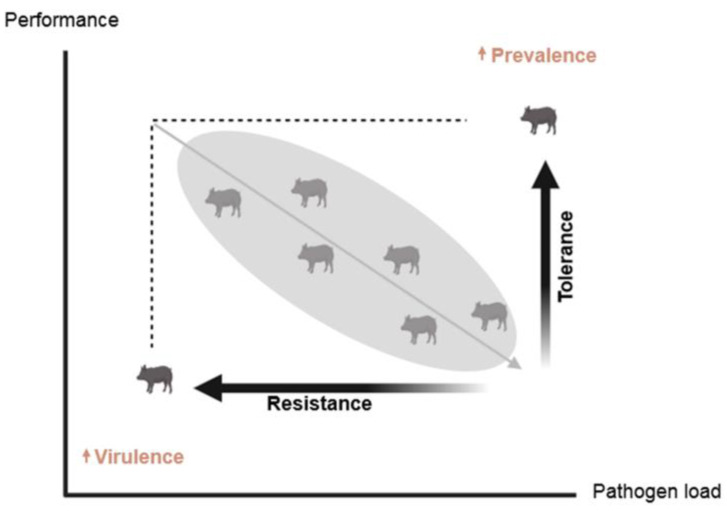
Illustration of the concepts of resistance and tolerance. Increasing resistance may increase pathogen virulence, while improving tolerance may increase pathogen prevalence and transmission.

Other concepts related to resilience, specifically disease resilience, are resistance and tolerance (Box 1, Figure 2). Disease **resistance** refers to the ability of an animal to control the pathogen load when challenged with a pathogen [30]. In contrast, disease **tolerance** indicates the ability of an animal to be minimally affected by a pathogen load [31]. Resistance helps to limit the pathogen load and consequently the transmission of the pathogen, whereas tolerance helps to lessen the clinical signs of disease, contributing to overall animal welfare without affecting the transmission of the pathogen at the population level. It is important to note that since the pathogen burden is difficult and costly to measure at the individual level in large populations, the concept of resilience has usually been used instead of resistance and tolerance [31]. Research revealed that selection for improved resistance may adversely affect pathogens’ evolution because they may evolve and increase their virulence due to high selection pressure [32,33]. Furthermore, selection for improved tolerance may increase the transmission of pathogens and consequently infection of susceptible animals [34]. Thus, from an epidemiological point of view, this selection approach may have negative effects on controlling diseases at the population level in the long term. Therefore, an alternative strategy is breeding animals for enhanced resilience since selection for resilience also improves animals’ resistance and tolerance [15].

## 3. Quantifying Animal Resilience

**Figure 3:**
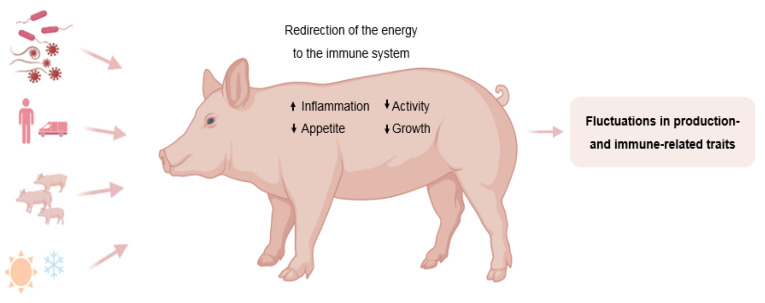
Challenges lead to deviations in production- and immune-related traits. Created with BioRender.com.

The lack of consensus in defining resilience has also limited agreement on a straightforward quantifying method, which is a crucial prior to implementing a selection program for any trait. Resilience is a complex trait that depends on multiple biological processes [20]. Under challenging conditions, trade-offs occur between these basic physiological functions (Figure 3). Immune functions compete with other energy-demanding processes such as growth [26], and energy may therefore be diverted towards the immune system to support an animal’s response to a challenge instead of expression of its production potential [26,35]. For example, one of the first consequences of activation of the immune system and inflammation is to suppress appetite and consequently reduce productivity [35]. Examples of these trade-offs between growth and immunity have been demonstrated in layer chickens [36], sheep [37], and pigs [38]. In this sense, perturbations lead to deviations in the basic production- and immune-related traits of challenged animals. Therefore, in recent years, researchers have investigated the use of these deviations as proxies to quantify resilience in various species. Such metrics are termed “**resilience indicators**”.

**Resilience indicators** may be defined as easily measurable **phenotypes** that capture and reflect disturbances caused by **perturbation(s)** that may be infectious or non-infectious. Several phenotypes that reflect such disturbances have been used, such as deviations in production traits, immunocompetence, or an animal’s activity and behavior. As mentioned in the former section, perturbations may vary from a specific pathogen to all the challenges that may occur during an experiment [39]. Therefore, depending on both the analyzed phenotype and the perturbation, resilience indicators capture either resilience to a specific disease or an animal’s general resilience. Examples of some resilience indicators proposed in previous publications are given in Table 1. Resilience indicators were elaborated based on deviations in production traits such as milk yield in cattle [40,41,42,43,44], litter size in rabbits [45], feed efficiency in pigs [46,47,48], egg production in hens [49,50], body weight in cattle [40], pigs [46,51], chicken [52], and fish [53], and wool fiber diameter profiles in sheep [54]. Other resilience indicators were elaborated based on immune-related traits such as plasma protein levels [51,55], complete blood counts [56], natural [57] or total [58] antibody titers, and total IgG and IgM production [57,59]. Recently, fluctuations in activity data such as individual activity in pigs [60], migration strategies in shorebirds [61], and daily step counts in cattle [62] were also investigated to quantify resilience. Moreover, biomarkers of stress responses, such as cortisol, ACTH, and DHEA, have been investigated, among others, as indicators of the host response to challenges and resilience [63]. Table 1 summarizes resilience indicators that were clearly assigned to the concept of resilience by their authors or in follow-up work. Specific indicators for robustness, resistance, or tolerance were considered beyond the scope of our review, although sometimes, these concepts were interchangeably used with resilience. Notably, other traits such as the residual variance of litter size [64] and birth weight [65] in rabbits, litter size [66] and body weight [67] in pigs, weight gain [68] and body weight [69] in mice, body weight in chickens [70], and body weight in snails [71] were used as indicators of uniformity and canalization. These latter variables may be also considered as proxies for animal resilience. However, in many of these examples, the external perturbation was not clearly defined. Instead, all the challenging situations that the animals could encounter during their productive lives were gathered.

In general, resilience indicators are elaborated from phenotypes that reflect disturbances. Therefore, the key in this section is “*phenotyping*”. In the era of big data and precision technology, some intensive farms have access to longitudinal data such as milk yield, individual activity, egg production, or body weight, where many records can be registered. In previous years, researchers evaluated longitudinal data to quantify resilience [39] and suggested that resilient animals show fewer fluctuations than susceptible ones [19]. The main condition for applying this strategy is to have multiple accurate records of the same phenotype. Using simulated data, the minimum number and time distance between data points have been discussed in detail [74]. The drawback of this approach is the time needed to collect data. In an ideal world, it would be highly desirable to have a simple-to-measure phenotype that could be measured in young unchallenged animals and could predict an animal’s response to subsequent challenges. Such phenotypes are still elusive, but maybe, the genotypes of specific molecular markers could fit the same purpose.

## 4. Genetic Background of Resilience

**Figure 4:**
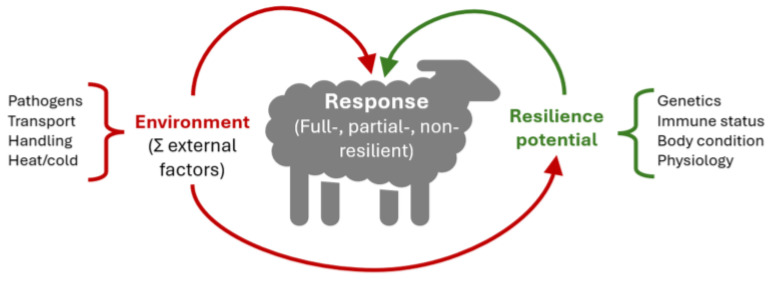
The environment can have a two-fold role in the activation of an animal’s resilience response.

The genetic basis of resilience is thoroughly evidenced, suggesting that animals are more resilient or susceptible to challenges depending on their genetics and, very likely, epigenetic labels. Indeed, several studies show that early exposure of animals to a diverse (enriched) environment improves their capacity to adapt to fluctuations later in life [75,76]. Therefore, the environment can have a two-fold role in the activation of resilience responses in the animals: first, as a modulator of the inherent capacity of an animal, and second, as a source of perturbations that can trigger a resilience response (Figure 4).

Initially, research on the genetic background of resilience was limited to the study of resilience to specific diseases. However, in the last decade, the elaboration of indicators for animals’ general resilience made the study of its genetic background possible. In the following section, we will provide an overview of the genetic background of (i) disease resilience (mainly in pigs) and (ii) animals’ general resilience.

### 4.1. Disease Resilience

#### 4.1.1. Pathogen-Specific Disease Resilience

The genetic component of the host response to diseases has been confirmed in various species, such as porcine reproductive and respiratory syndrome (PRRS) in pigs [77], mastitis [78,79], *Theileria parva* infection [80] in cattle, and influenza virus infection in chicken [81,82].

A few DNA markers with a large impact on disease resistance or tolerance have been described. In pigs, susceptibility to enterotoxigenic *Escherichia coli* (ETEC) F18, which is responsible for post-weaning diarrhea, was found to be regulated by a single nucleotide polymorphism (SNP; rs335979375, T>C) within the fucosyltransferase 1 (*FUT1*) gene [83,84]. The presence of the T allele is associated with improved survivability of pigs (from weaning to market) even in the absence of overt diarrheal outbreaks [85]. In a related manner, resistance to ETEC F4ab/ac infection has been located in pig chromosome 13 and can be typed with high accuracy with the tag marker CHCF1/rs340488770 [86,87]. These markers segregate in a number of western and Asian commercial lines [86,88,89,90], with variable allelic frequencies. However, the penetrance of the markers might still depend on the virulence and infective dose of the ETEC strain, with some pigs developing mild diarrhea despite having the resistant genotype [87], highlighting the multifactorial nature of this disease. Examples of pathogen-specific tolerance in other species are restricted to one or few breeds. For instance, the innate genetic tolerance of East African cattle to tick-borne *Theileria parva* infection has been attributed to a stop-gained mutation (rs209669442 C>T) in the FAS-associated factor 1 (*FAF1*) gene [80]. This variant is present at a low allele frequency in the Boran and Shorthorn Zebu breeds. The presence of the tolerant T allele improves the survival time and rate of infected cows probably by impairing replication of infected lymphocytes. Again, penetrance of the genotype is not complete and likely dependent on the infective dose and other health constraints in the animal.

Despite the above, polymorphisms with large effects on resilience to infections are rare as most diseases are polygenic and depend on many variants with small effects. For instance, much research has focused on studying the genetic determinism of the host response to PRRS virus infection in pigs [77] (Table 2).

A major Quantitative Trait Locus (QTL) within pig chromosome (SSC) 4 explained up to 15% of the genetic variance of viremia levels during a PRRS outbreak [91]. At first, the genetic marker rs80800372, also known as WUR10000125, at the 3′ UTR region of the guanylate binding protein (GBP) 1 (*GBP1*) gene was used as a tag variant and was validated across different genetic lines challenged with PRRSV-2 [91,102] and PRRSV-1 [103] strains. All of these studies reported that the alternative G allele is favorable for PRRS resilience. Subsequent studies attributed the causality of this QTL to the genetic marker rs340943904 at the *GBP5* gene [92]. Within the same gene family cluster, other point mutations such as rs708662877 at *GBP1* [101,104] and rs322187731 at *GBP6* [94,104] were also suggested to be associated with the host response to PRRS. The importance of this genomic region on the first-line response to PRRS is beyond doubt, but the relative importance of individual markers within might depend on the rate of linkage disequilibrium across them, which is estimated to be in the range of r^2^ = 0.6−1.0 in many commercial lines [92,104,105,106] and to be as low as r^2^ = 0.21 in Korean native pigs [107] or 0.13 in Italian breeds [108]. Another important candidate gene for the host response to PRRS is the cysteine-rich scavenger receptor (*CD163*), which encodes a sufficient and necessary cellular receptor for the PRRS virus. Knockout of *CD163* led to complete resistance against PRRS virus infection [109]. In addition, polymorphisms within this gene were found to be associated with the host response to PRRS virus infection, such as viremia, immune-related traits, and the probability of abortion, in infected sows [93,96,110,111]. Another cellular receptor associated with the host response to PRRS is sialic acid binding Ig-like lectin 1 (*CD169*). Although there is controversial information about whether *CD169* is required for PRRS infection or not [112,113], several polymorphisms within this gene were associated with the host response to PRRS [95,96,110]. Moreover, a polymorphism at the promoter of the ubiquitin-specific protease 18 (*USP18*) was found to increase the expression of this gene and inhibit PRRS virus replication in Chinese pig breeds [98,114]. Notably, this polymorphism did not segregate in Duroc, Yorkshire, and Landrace pigs [93,98]. In addition, genomic regions and variants within the Major Histocompatibility Complex (*MHC*) locus were associated with the antibody response [100,115,116] and reproductive performance [97] during PRRS outbreaks. Many other polymorphisms were associated with PRRS infection and outcomes, such as the myxovirus resistance protein 1 (*MX1*) [93,101], serum and glucocorticoid-regulated kinase 1 (*SGK1*) [97], and the regulator of G protein signaling 16 (*RGS16*) [110]. Each one of these genes contributes to a more effective response to infection. However, it must be noted that, except for the markers on the GBP cluster, other markers have only been explored in one or very few experiments, and their resilience effects cannot be considered validated. Therefore, caution must still be exercised with respect to their current applicability in breeding programs.

The pig genetic background could affect the outcomes of other viral diseases, such as classical (CSF) and African (ASF) swine fever. Overexpression of the porcine radical S-adenosyl methionine domain containing 2 (*RSAD2*) gene inhibited the replication of the CSF virus and reduced the infection in vitro [117]. Additionally, knock-out of Poly (rC)-binding protein 1 (*PCB1*) inhibited CSF infection [118]. Moreover, a genome-wide association study detected genomic regions associated with the antibody response after CSF vaccination [119]. Regarding ASF, knowledge about how host genetics regulate the infection is controversial. Sánchez-Torres et al. [120] suggested that *CD163* regulates the host response to ASF virus infection. In contrast, neither *CD163* overexpression [121] nor knock-out [122] were associated with resilience towards the infection. However, point mutations within the *RELA* gene reduced ASF virus infection and lessened its clinical severity [123]. Similarly, the role of the host response was also evidenced for other diseases, for instance, the porcine epidemic diarrhea virus [124,125], transmissible gastroenteritis virus [126,127], and porcine delta coronavirus [127,128]. Previous reviews have provided in-depth details for the genetic backgrounds of responses to avian oncovirus, such as Rous sarcoma, Marek disease, and the avian leukosis virus [129], and tuberculosis [130] and paratuberculosis [131] in cattle. Many of these reports highlight the importance of the *MHC* locus in initiating effective antigen responses, which influence the duration and magnitude of subsequent antibody production.

#### 4.1.2. Polymicrobial Disease Resilience

These former studies were limited to the genetic basis of resilience to specific diseases. A recent experiment demonstrated the genetic background of polymicrobial disease resilience in pigs using a multifactorial challenge, including bacterial and viral diseases [48]. Simultaneous challenges with multiple pathogens are a common situation under field conditions. Therefore, this approach can help to identify resilience responses, allowing fast translation to daily practice. Deviations in feed intake and feeding time were used as indicators for polymicrobial disease resilience in pigs. These indicators were genetically controlled with low-to-moderate heritability estimates ranging from 0.15 to 0.26 [48,132]. Further, within this experiment, several indicators for polymicrobial disease resilience were suggested and were reported to be under genetic control, namely, natural antibody titers [57], complete blood counts [56], and feeding and drinking traits [133]. Moreover, association analyses for the resilience indicators elaborated in this natural polymicrobial disease challenge identified several associated genomic regions [134], which can be taken as a proof of concept that breeding for resilience to a broad range of pathogens could be possible.

### 4.2. General Resilience

Deciphering the genetic background of disease resilience is important. However, animals are confronted not only by pathogens during their productive lives. Animals face a wide range of perturbations (biotic and abiotic) under field conditions [135]. Thus, it is also important to investigate the genetic determinism of general resilience. There is scarce information about this field of study in the scientific literature owing to the absence of a gold standard quantifying method for general resilience. In recent years, novel indicators have been used as proxies for general resilience and have enabled demonstration of its genetic component (Table 3). Heritability estimates of resilience indicators were low to moderate, indicating that general resilience is under genetic control but also largely dependent on environmental variation to trigger the penetrance of the genotype (Table 3). Moreover, the genetic background of residual variance, which was recently suggested to be an indicator of resilience, has been evidenced in pigs [66,67], mice [68], chicken [70], and cattle [136]. This is certainly a promising indicator that will need to be studied in more detail in the future to characterize critical parameters such as heritability.

Given the lack of consensus in defining general resilience, to date, the molecular mechanisms that underlie this trait are not well defined. Some genome-wide association studies (GWASs) have been carried out to elucidate the mechanisms underlying animals’ general resilience, including GWASs for the environmental variance of litter size in rabbits [138]; deviations in expected growth, increment of the acute-phase protein haptoglobin [139], and the resilience response after a challenge [140] in pigs; and fluctuations in egg production in chickens [50]. In rabbits, Casto-Rebollo et al. [138] identified four genomic regions associated with the residual variance of litter size, which harbor candidate genes related to immune and stress responses such as dedicator of cytokinesis (*DOCK2*), histone deacetylase 9 (*HDAC9*), and integrin beta-8 (*ITGB8*). In pigs, association analyses for two resilience indicators, deviation from the expected body weight and increases in haptoglobin, identified eight genomic regions at pig chromosomes 2, 8, 9, 11, and 13 [139]. These associated regions harbored potential candidate genes involved in the immune response pathway such as prostaglandin D2 receptor 2 (*PTGDR2*), ribonuclease L (*RNASEL*), and myeloid differentiation primary response gene 88 (*MYD88*), as well as growth factor receptor bound protein 10 (*GRB10*). In chickens, Doekes et al. [50] did not find relevant associations with fluctuations in egg production, corroborating the absence of a major gene regulating resilience. Taken together, association analyses for general resilience have not detected any associated variant or genomic region with a major effect. The identified candidate genes were mainly involved in the immune response, stress responses, and signaling and growth pathways. The overlap between the candidate genes proposed by these studies is minimal, and the roles of individual markers lack validation. Yet, taken together, these findings support the polygenic nature and inheritance of resilience, as well as the strong relationship between resilience, immunity, and growth.

### 4.3. Resilience and Immunity

The candidate genes and variants associated with resilience vary among studies depending on the described resilience indicators and the studied population. Nevertheless, all research findings have highlighted that resilience is a complex and polygenic trait that depends on various physiological processes [50,138,139,140]. In general, candidate genes for resilience are mainly involved in the immune response pathway, corroborating the relationship between resilience and immunity, which has been emphasized by several studies. For instance, in rabbits, findings from a divergent selection experiment on the environmental variance of litter size as an indicator of general resilience [64] reported that the low line (low environmental variance of litter size) was less susceptible to stress and infections than the high line (high environmental variance of litter size) [45,141]. The low line showed lower basal levels of cortisol and C-reactive protein than the high line, denoting a lower pro-inflammatory state than that of the high line. When faced by challenging situations, such as the first delivery, the low line showed higher white blood leukocyte counts, higher C-reactive protein levels, and lower tumor necrosis factor-alpha levels than the high line, indicating the ability of the low line to cope with stressful situations [141]. Colditz and Hine [20] suggested that a high adaptative immune response may be associated with better resilience to disease challenges. In addition, Broom and Kogut [35] reported that an optimal scenario to maintain an effective response to infections relies on a strong inflammatory reaction, followed by an effective anti-inflammatory response. In fact, the resilience response relies on two processes: coping with the challenge(s) and bouncing back to normal functioning. In the first phase, resilient animals resist challenges by initiating strong inflammatory reactions. However, during this pro-inflammatory phase, immune functions compete with other energy-demanding processes, and therefore, they adversely affect basic physiological functions. Thus, in the second phase of the resilience response, animals must develop an anti-inflammatory response to attenuate and overcome the adverse effects of inflammation and bounce back to normal functioning [35,142].

Resilience has also been associated with the growth pathway. As mentioned previously, the inflammatory response initiated after challenging conditions adversely affects growth and productivity [35]. Therefore, this association between resilience and growth may be an indirect consequence of the association between resilience and the immune response and initiation of the inflammatory response under stressful conditions. However, further analyses are needed to validate this hypothesis.

## 5. Incorporation of Resilience Indicators in Selection Schemes

Since the genetic component of animal resilience has been well evidenced in various species (previous section), selective breeding was suggested as a strategy to improve this trait. Selective breeding for enhanced resilience could enhance an animal’s ability to cope with challenges, resulting in more robust phenotypes [20,143]. Therefore, it would benefit (i) animals through improvements in their welfare and well-being; (ii) farmers through reductions in economic losses and increases in profitability; and in the long term, (iii) the environment by increasing sustainability and the efficiency of production farms and through possible reductions in therapeutic treatments (mainly antimicrobials) with a high one-health impact. The main advantage of selective breeding is that the generated genetic gain is consistent and cumulative across generations. However, it cannot be overlooked that additional disease control procedures, such as vaccination, adequate herd management, and biosecurity measures, are essential to maximize the expression of the genetic gain in livestock.

### 5.1. Importance of the Association Between Resilience and Production

It is important to point out that there may be antagonism between resilience and production [10], and breeding for an enhanced specific immune response may adversely affect general resilience [144]. Thus, it is crucial to assess associations between economically important traits and resilience indicators prior to their implementation in breeding programs. Some research has been conducted regarding the effects of candidate markers for resilience to PRRS [106,108,145] and ETEC F4ab/ac infection [86] on pig production traits under non-challenging conditions. However, as far as we know, to date, the effects of general resilience indicators on production traits have not been estimated under non-challenging conditions.

### 5.2. Breeding for Resilience at the Experimental Level

There has been increasing interest in improving resilience through genetic selection [12]. Resilience indicators—which capture disturbances caused by stressors, are genetically controlled, and present variability within a population—may be considered in breeding programs. Simulation studies showed that including resilience indicators in breeding goals may improve resilience and reduce the probability that disturbances influence the performance of pigs and cattle [19]. In the absence of a direct quantification method for resilience, different approaches have been explored to enhance animal responses to disturbances, such as (i) selection for enhanced immunocompetence and health and (ii) selection for higher uniformity and lower residual variance. To the best of our knowledge, most of these strategies were executed under experimental conditions and have not yet been implemented in breeding schemes. In pigs, for example, a divergent selection experiment for general immune responsiveness was conducted by [146] in order to select animals for broad immune responses. Yorkshire pigs were classified according to low or high immune responses based on fourteen innate and adaptive immune traits. Pigs from the high-immune response line showed improved health, responses to vaccination, and growth compared to pigs from the low-immune response line [147,148]. In dairy cattle, the incidence of diseases, such as mastitis, displaced abomasum, and retained fetal membranes, was higher in low-immune response lines than in high-immune response lines [149]. These results suggested that selection for general immune responsiveness resulted in positively correlated results for the animal response to infectious diseases and growth traits. In rabbits, a divergent selection experiment was conducted for the environmental variance of litter size [64]. The line with high environmental variance of litter size showed higher susceptibility to challenges than the line with low environmental variance of litter size [45,141]. This selection experiment suggests that breeding for higher uniformity (low environmental variance) leads to more resilient animals [45]. In line with these results, another divergent selection experiment for the environmental variance of birth weight in rabbits revealed that selection for low environmental variance of birth weight resulted in lower pre-weaning mortality [65].

### 5.3. Breeding for Resilience at the Commercial Level

As far as we know, resilience per se has not been completely included in the breeding goal of commercial lines. Recently, some pig breeding companies have been exploring the implementation of disease resilience in their breeding programs. For instance, *Topigs Norsvin* recently included the genetic marker WUR10000125 for the host response to PRRS [150]. Furthermore, in dairy cattle, some health-related traits have been considered in breeding, such as udder health [151] and claw health [152]. Instead, several companies are considering robustness in their breeding goal using a diverse range of indicators. For instance, *Danish Genetics* implemented robustness in the breeding goal of Yorkshire, Landrace, and Duroc pigs. This company selects for enhanced robustness by selecting for longevity and strength (i.e., leg position score) in Yorkshire and Landrace pigs and for strength and survivability in Duroc pigs [153]. Additionally, the *Pig Improvement Company* (PIC) reported the implementation of robustness in their breeding goal by including pig soundness (i.e., leg conformation) and livability in pig breeding programs [154,155]. Similarly, *Topigs Norsvin* reported selecting pigs for enhanced robustness by considering proxy traits such as vitality and litter uniformity for piglets and mortality and leg structure at the finishing period [156].

### 5.4. Future Trends to Improve Resilience

The evidence of a genetic component of resilience and the identification of associated candidate genes and variants enable marker-assisted selection. This strategy is efficient when traits are controlled by variants with major effects [157], such as the *FAF1* marker for *Theileria parva* tolerance in cattle [80], the *FUT1* [83,84] and CHCF1 [86] markers for ETEC infection in pigs, or even the WUR1000125 marker, which explains up to 15% of the genetic variance of the host response to PRRS [91]. However, genomic selection seems promising when traits of interest are expensive to measure, present low heritability, and are polygenic, such as resilience. Genomic selection takes into account the effects of all the genotyped variants in a population [157,158]. Therefore, variants with minor effects and associated variants, which were not detected due to detection power issues, are all considered to predict the genetic merit of individuals. Moreover, genomic selection enables evaluation of the genetic merit of healthy animals for traits that are typically collected from infected animals under challenging conditions. Another possible technology to improve animal resilience, especially disease resilience and the host response to infections, is gene editing [159]. This strategy, for instance, has led to the commercialization of heat resilient cattle in the USA and Brazil. A modification of the prolactin receptor gene in these animals results in shorter hair, which reduces insulation to conductive and convective heat loss in the slick haircoat [160]. In cattle, knock-in of natural resistance-associated macrophage protein 1 (*NRAMP1*) increased resistance against tuberculosis [161]. While in chicken, the knock-out of the retrovirus receptor *tva* led to resistance against avian leukosis virus infection [162]. In pigs, the most studied example is *CD163* for the host response to PRRS, as its knock-out resulted in resilience of pigs against PRRS virus infection [109]. Recently, the PIC breeding company reported exploring gene editing to enhance the host response to PRRS [163]. Yet, an important question is whether society would accept consuming gene-edited products. This is a question being raised in Europe for edited plants. A proposal to deregulate new genomic techniques is being studied by the European Commission as part of a legal package promoting the sustainable use of natural resources [164]. However, there is less certainty regarding the future use of gene-edited animals. In addition, although gene editing is a promising strategy, it might not be adequate for general resilience, which is a polygenic trait with no major associated variants to date. Hence, a possible strategy to improve general resilience might rely on including candidate genetic markers in breeding programs. In the absence of a straightforward quantifying method for general resilience, this approach may be conducted as follows:

i. Estimate **resilience indicators** using deviations in production, activity, and/or immune traits.
ii. Validate the elaborated resilience indicators by estimating their **association with resilient phenotypes** such as mortality, treatment rate, or incidence of infections.
iii. Investigate the genetic background of general resilience through genome-wide association studies to identify **associated genetic markers** with the elaborated resilience indicators.
iv. **Validate** the most significant and functionally important genetic markers with larger datasets, with different lines, and, if possible, under different challenging situations.
v. Estimate the association between the validated genetic markers and economically important traits such as **production or reproductive performance**.
vi. Include the validated variants for general resilience with no (or positive) effects on production traits in breeding programs to improve general resilience without adversely affecting production (productive resilience).

## 6. Conclusions

In this review, we underlined the importance of breeding for resilience in production systems, along with proper production strategies and biosecurity conditions. Resilience is an important trait since it would benefit animals (better welfare and well-being), producers (reduced economic losses and increased profitability), the environment (better sustainability), and the use of antimicrobials with a one-health approach (reduced use in resilient animals). Nevertheless, the study of resilience has been limited due to the absence of a gold-standard quantifying method. Therefore, resilience indicators have been elaborated to quantify animal resilience. Throughout the years, the focus has been on studying specific disease resilience and immune responsiveness. However, animals are exposed to not only pathogens in commercial farms but also a wide range of challenges and perturbations. Moreover, breeding for specific disease resilience may be beneficial in the short term, but pathogens evolve. Hence, it would be essential to advance our knowledge about an animal’s general resilience, unravel its genetic background, and, in the future, test breeding animals for enhanced general resilience.

## Figures and Tables

**Table 1:** Summary of some resilience indicators in livestock species.

Proxy	Resilience Indicator	Species	Reference
Production trait	Deviation ^1^ in body weight	Cattle	[40]
	Deviation in milk yield	Cattle	[42,43,44,55,72]
	Deviation in egg production	Chicken	[49,50]
	Deviation in body weight	Chicken	[52]
	Deviation in body weight	Tilapia	[53]
	Deviation in litter size	Rabbits	[45,64]
	Deviation in body weight	Pigs	[46,51]
	Deviation in feed efficiency	Pigs	[46,47,48]
	Deviation in wool fiber diameter	Sheep	[54]
Immunophenotype	Plasma protein levels	Pigs	[51,55]
	Complete blood count	Pigs	[56]
	Antibody titers	Pigs	[57,58]
Biomarkers	Hair DHEA concentration	Pigs	[73]
Activity data	Deviation in individual activity	Pigs	[60]
	Migration strategy	Shorebird	[61]
	Deviation in daily step count	Cattle	[62]

^1^ Deviation refers to any indicator used to quantify perturbations after challenge in production traits, immunophenotypes, and activity.

**Table 2:** Candidate genetic markers for the host response to porcine reproductive and respiratory syndrome.

Gene	Marker	Position ^1^	Polymorphism	Reference
***GBP1***	rs80800372	4:127441677	A>G	[91]
***GBP5***	rs340943904	4:127301202	G>T	[92,93]
***GBP6***	rs322187731	4:127190259	A>G	[94]
***CD163***	rs1107556229	5:63325006	G>A	[93]
***CD169***	rs345830287	17:32003653	G>A	[95]
***CD169***	rs342517982	17:32007161	C>A	[96]
***CD169***	rs323502146	17:32015498	C>T	[96]
***SGK1***	rs338508371	1:29753070	C>A	[97]
***TAP1***	rs80928141	7:25068055	C>T	[97]
***USP18***	rs330732437	5:70181788	G>A	[98]
***MHC***	rs80795088	7:25049757	A>C	[99]
***MHC***	rs80900036	7:24217931	T>C	[100]
***MX1***	−547ins+275	13:204845962	275 bp insertion	[93,101]

^1^ Position in the pig genome assembly *Sscrofa 11.1* (chromosome: position in bp).

**Table 3:** Heritability estimates (h^2^) of some indicators of general resilience.

Species	Resilience Indicators	h^2^	Reference
Cattle	Deviations in daily step counts	0.01 to 0.15	[62]
	Deviations in milk yield records	0.01 to 0.15	[41]
		0.004 to 0.25	[44]
		0.01 to 0.24	[43]
		0.06 to 0.10	[42]
Chickens	Deviations in body weight	0.09 to 0.11	[52]
	Deviations in egg production	0.01 to 0.21	[49]
Pigs	Deviations in body weight and haptoglobin	0.16 to 0.33	[51]
	Deviations in feed efficiency	0.31 to 0.40	[47]
Goats	Fecal egg count and somatic cell score	0.07 to 0.21	[137]
Sheep	Fecal egg count and somatic cell score	0.13 to 0.14	[137]
	Profiles of pool fiber diameter	0.01 to 0.18	[54]
Rabbits	Residual variance of litter size	0.08	[64]
Tilapia	Deviations in body weight	0.10 to 0.12	[53]

## Data Availability

Not applicable.
